# Carcinomes hépatocellulaires en milieu africain burkinabè: contribution de l’échographie à propos de 58 cas

**Published:** 2010-11-04

**Authors:** Zakari Nikièma, Appolinaire Sawadogo, Carole Gilberte Kyelem, Rabiou Cissé

**Affiliations:** 1 Service d'Imagerie Médicale, Centre Hospitalier Universitaire Sourô Sanou, Bobo-Dioulasso, Burkina Faso; 2 Département de Médecine, Centre Hospitalier Universitaire Sourô Sanou, Bobo-Dioulasso, Burkina Faso; 3 Service d'Imagerie Médicale, Centre Hospitalier Universitaire Yalgado Ouédraogo, Ouagadougou Burkina Faso

**Keywords:** Carcinome hépatocellulaire, échographie, Alpha-foetoprotéine, Burkina

## Abstract

**Introduction:**

Décrire les différents aspects échographiques de carcinome hépatocellulaire (CHC) et préciser l’apport de l’échographie dans le diagnostic des CHC dans le contexte burkinabè.

**Méthodes:**

U+00C9tude prospective descriptive de 12 mois, réalisée dans les services de médecine interne et d’imagerie médicale du CHU Souro Sanou de Bobo Dioulasso, portant sur 58 patients ayant un CHC. Tous les patients ont bénéficié d’une exploration échographique abdominale et dans certains cas d’un dosage du taux d’alpha-foetoprotéine et d’un examen histologique hépatique. Les caractères échographiques des tumeurs et les signes extra tumoraux associés ont été notés.

**Résultats:**

Cinquante huit patients d’âge moyen de 45,6 ±12,5 ans (extrêmes 22-77 ans), avec une nette prédominance masculine (n=44) étaient inclus dans l’étude. Le retard diagnostic était de plus de 3 mois dans 50,7%. L’échographie décrivait des grosses tumeurs (69% des cas), multinodulaires (67,2%), à  majorité hyperéchogènes (70,7%). Cinq formes pseudo suppuratives étaient notées (8,7%). L’ascite (60,3% des cas) et la thrombose portale (56,8%) étaient les signes extra-tumoraux les plus fréquents. La thrombose cave inférieure (n=1) était rare. L’alpha-foetoprotéine était significativement élevée chez 10 patients (43,5%). L’examen histologique (n=20) décrivait un CHC chez 18 patients (90%).

**Conclusion:**

Au Burkina Faso, le CHC est multinodulaire, hyperéchogène et de grosse taille avec une forme particulière pseudo abcédée. L’imagerie échographique est un examen complémentaire indispensable décrivant des aspects presque pathognomoniques du CHC. Toutefois, son association à l’histologie est plus utile que le dosage de l’alpha-foetoprotéine dans le diagnostic des CHC.

## Introduction

Le carcinome hépatocellulaire (CHC) est la  tumeur primitive du foie la plus fréquente dans le monde. La population  à risque est constituée des sujets atteints d’hépatopathie chronique au stade de fibrose extensive ou de cirrhose. L’incidence est globalement de 11 sur 100 000 hommes et de 1,5 sur 100000 femmes [1]. Cette tumeur est de distribution géographique inhomogène. En effet 80% des patients porteurs de CHC sont originaires de pays en voie de développement. Son incidence est de plus de 20 cas sur 100.000 habitants en Asie du Sud et en Afrique subsaharienne qui sont des zones endémiques de l'infection par le virus de l’hépatite B [1,2]. L’étiologie du CHC est dominée par les virus des hépatites B et C, la consommation massive d’alcool et la stéatohépatite non alcoolique [1].

Dans les pays du nord, le CHC est habituellement de  découverte précoce dans le cadre du suivi d’une cirrhose et touche principalement les sujets âgés [1]. Par contre, en Afrique noire, le CHC reste encore une affection de découverte tardive au stade de développement tumoral massif. Au Burkina Faso, le problème posé par le CHC est connu depuis de longues années et en 1974, sa fréquence était de 1,73% de l’ensemble des hospitalisés en zone rurale [3].

En Afrique, la majorité des études se sont intéressées aux aspects épidémio-cliniques, thérapeutiques ou évolutifs [4,5]. Par contre, peu d'études ont décrit les aspects échographiques des CHC [6]. En effet, l'application de l'imagerie diagnostique est d'une importance fondamentale dans le dépistage, le diagnostic et le traitement du CHC. Parmi les options d'imagerie diagnostique, l'échographie a un rôle de premier plan. De part ses caractères intrinsèques, elle permet une imagerie en temps réel d’une approche diagnostique précoce du CHC [7].

Le but de cette étude était de décrire les aspects échographiques de CHC et de préciser l’apport de l’échographie dans le diagnostic des CHC dans le contexte burkinabè.

## Méthodes

Il s’agit d’une étude prospective  descriptive de janvier 2008 à décembre 2009,  réalisée dans les services de médecine interne et d’imagerie médicale du CHU Sourô Sanou de Bobo Dioulasso, portant sur 58 cas de CHC.

Ont été inclus les patients de plus de 18 ans suspects de tumeur hépatique et ayant bénéficié d’une exploration échographique, d’un dosage du taux d’alphafoetoprotéine, d’une recherche sérologique de hépatite B (AgHBs) et C (Ac antiVHC), et d’un examen histologique hépatique. Le diagnostic était basé sur un taux élevé de l'alphafoetoprotéine (>500 ng/ml), sur les caractères échographiques des tumeurs et/ou sur la preuve histologique de CHC.

L’examen échographique a été réalisé à l’aide d’un échographe Logic 200 de marque General Electric muni d’une sonde de 3,5 MHZ et de 7,5 MHz. Il a consisté en une analyse abdomino-pelvienne par voie antérieure après un jeûn d’au moins 12 heures. L'analyse échographique a recherché les caractères des tumeurs notamment leur nombre, leur forme, leur taille [8]. Il s’agissait d’une petite tumeur, si son plus grand diamètre était inférieur à 5 cm et d’une grosse tumeur si le diamètre était supérieur ou égal à 5 cm. Lorsque les nodules étaient multiples, le diamètre considéré était celui du plus gros nodule. Les formes diffuses ou infiltrantes à limites imprécises ont été considérées d'emblée comme de grosses tumeurs. L'échogénicité des tumeurs se définissait en hyperéchogène, isoéchogène, hypoéchogène, liquidienne ou mixte. L'exploration échographique a également noté les lésions extra-tumorales associées.

La ponction hépatique était effectuée à l’aiguille fine sous guidage échographique en l’absence de contre-indications. Après la ponction, un contrôle échographique était effectué à la recherche de signes d’hémorragie patente.

## Résultats

### Caractéristiques épidémiologiques des patients

L’âge moyen des patients était de 45,6±12,5 ans  avec des extrêmes de 22 ans et 77 ans. Une nette prédominance masculine a été notée avec 75,7% d’hommes et 24,3% de femmes.

### Antécédents et motifs de consultation

Les antécédents les plus fréquents étaient: l’ictère (36,1%), l’alcool (31,9%), et l’hémorragie digestive dans 26,4% des cas. Les douleurs abdominales (83,8%), l’altération de l’état général (79,7%), l’augmentation du volume de l’abdomen (74,3%) et la perception d’une masse abdominale (67,1%) étaient les principaux motifs de consultation.

### Données cliniques

Le début de la maladie pour la plupart des patients se situait entre un et trois mois avec 38,3% et pour 32,9%, il se situait au-delà de 6 mois. L’hépatomégalie était le principal signe retrouvé chez 86,5% des malades suivie de l’ascite (51,4%). Le liquide péritonéal était hémorragique dans 34% des cas et d’aspect citrin dans 66% des cas. Les autres signes étaient surtout l’ictère (35,1%) et les oedèmes des membres inférieurs (28,8%).

### Données biologiques et cytologiques

Le dosage de l’AFP a été effectué chez 23 patients. L’AFP était supérieure à 500ng/ml, significativement élevé chez 10 patients soit 43,5%. La recherche de l’AgHBs était positive dans 75,5% des cas. Les anticorps anti VHC étaient présents dans 13,6% des cas. La ponction échoguidée hépatique réalisée à l’aiguille fine (n=20) était contributive dans 18 cas (90%) et constituée uniquement de CHC.

### Imagerie échographique

L’échographie abdominale était effectuée chez 74 patients et une anomalie suspecte de néoplasie hépatique était décrite chez 58 d’entre eux, soit 78,3% des cas. On distingue: les anomalies hépatiques (tableau 1) et les anomalies extra-hépatiques (tableau 2).

**Figure 1:**
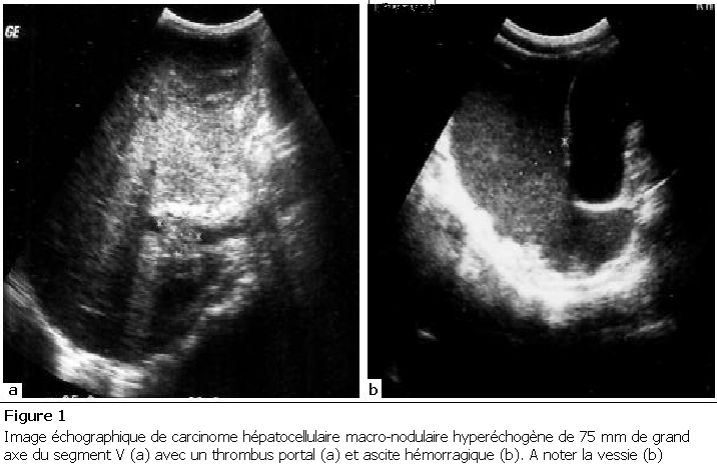
Image échographique de carcinome hépatocellulaire macro-nodulaire hyperéchogène de 75 mm de grand axe du segment V (a) avec un thrombus portal (a) et ascite hémorragique (b). A noter la vessie (b)

**Figure 2:**
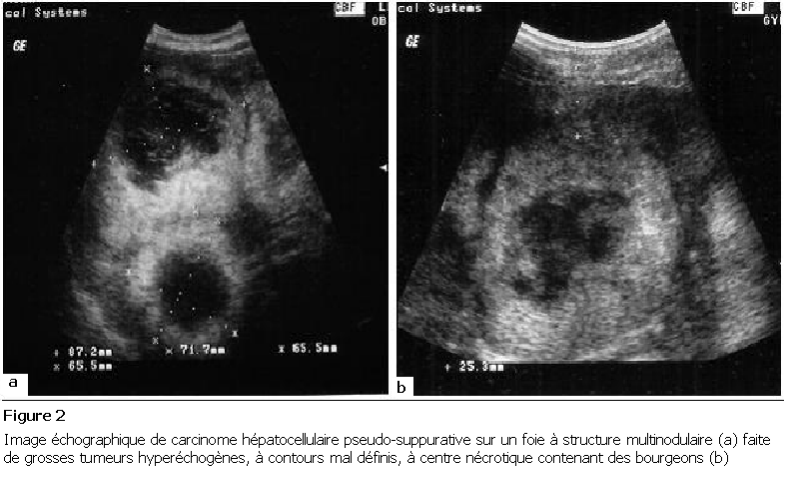
Image échographique de carcinome hépatocellulaire pseudo-suppurative sur un foie à structure multinodulaire (a) faite de grosses tumeurs hyperéchogènes, à contours mal définis, à centre nécrotique contenant des bourgeons (b)

**Figure 3:**
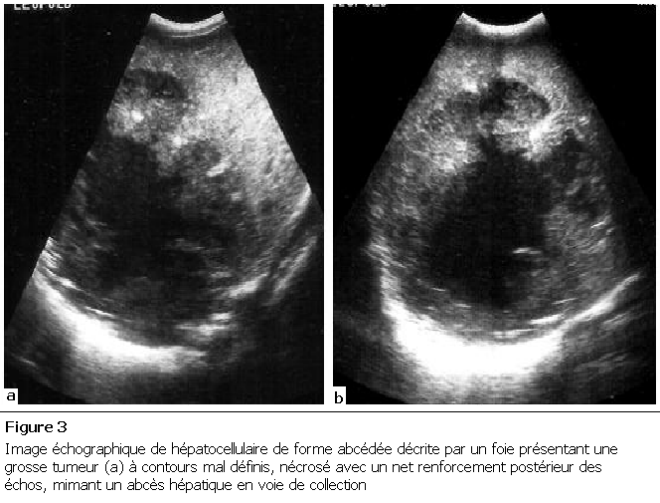
Image échographique de carcinome  hépatocellulaire de forme abcédée décrite sur un  foie présentant une grosse tumeur (a) à contours mal  définis, nécrosée avec un net renforcement  postérieur des échos, mimant un abcès hépatique en  voie de collection

La figure 1 décrit une image échographique de CHC de forme macro-nodulaire hyperéchogène. La figure 2 décrit une image de CHC de forme pseudo-suppurative et la figure 3 celle d’une forme abcédée.

## Discussion

L’exploration échographique a permis une approche diagnostique des  différents stades évolutifs des CHC dans notre contexte  burkinabè. En effet, notre étude retrouve des tumeurs surtout  nodulaires, hyperéchogènes, multiples et à majorité  de grande taille, caractères conformes à ceux retrouvés par  N’Gbesso en Côte d’Ivoire [6]. Au contraire dans les séries  occidentales, les CHC sont de petites lésions nodulaires de moins de 3  cm, isolées qui augmentent progressivement de taille et plus  fréquemment hypoéchogènes et homogènes [1,9,10].  L'un des arguments pour expliquer cette différence, est la  détection précoce et la petite taille des CHC observés dans  ces pays de haute technicité des moyens d’imagerie médicale, en  particulier dans la population à risque des cirrhotiques. Les CHC de  petite taille sont surtout hypoéchogènes dans 55,5% des cas et les  gros CHC surtout hyperéchogènes [9,10]. Il semblerait que beaucoup  de petits CHC hypoéchogènes au début, deviennent  secondairement de gros CHC Page number not for citation purposes 4  hyperéchogènes (36% des cas), et les rares petits CHC  hyperéchogènes gardent généralement leur aspect en  devenant de grosses tumeurs dans 4% des cas [9]. Ces auteurs concluent que  l’échogénicité des petits carcinomes  hépatocellulaires augmente avec la croissance de la tumeur, et reste  inchangée quand ils n'augmentent pas de taille [9,10].

Du fait de  l'absence de suivi évolutif et la découverte tardive de la  maladie, la majorité de nos CHC étaient de grosses tumeurs  hyperéchogènes et constituent une particularité du sujet  noir africain. Cependant, Cottone dans une étude italienne rapporte des  aspects échographiques de CHC superposables aux nôtres. Dans son  étude, les patients porteurs de CHC étaient à 80% AgHbs  positifs et à 17% alcooliques [9]. Ce contexte  épidémiologique est donc superposable à celui de notre  étude oU+00F9 nous avons noté 75,5% patients AgHbs positifs et 23  patients (31,9%) alcooliques avérés. Dans ce contexte  épidémiologique, l'âge jeune de nos patients de 45,6 ans et  la prédominance masculine sont superposables aux données de la  littérature africaine [4-6]. Ces résultats tendent à  conférer au CHC le statut de pathologie masculine. Cela pour plusieurs  raisons. La consommation d’alcool et la prévalence des hépatites  virales chroniques B et C plus élevées chez l’homme que chez la  femme seraient incriminées.

Dans notre étude, une forme  particulière de CHC est à souligner de même que dans la  série de N’Gbesso [6]. C’est la forme suppurative ou à type  d’abcès non collecté notée chez cinq de nos patients et qui  était étiquetée d’abcès amibien du foie. Elle n’est  pas signalée dans la plupart des séries occidentales [1,9,10].  Mais l’absence d’évolutivité clinique et échographique  orientent vers un caractère suspect et imposent la réalisation  d’explorations complémentaires tel le dosage de l'AFP, l’histologie ou la  cytologie après ponction écho-guidée. La technique est  reconnue par tous les auteurs, sans danger et fiable lorsqu'elle est  réalisée par un opérateur expérimenté et  à l'aiguille fine [11]. Renard n'a observé aucune  différence significative entre les résultats obtenus par l'analyse  cytologique et ceux obtenus par l'analyse histologique [12]. Toutefois,  l'histologie n'est requise que lorsque la nature de la lésion  hépatique est douteuse [11].

Maringhini qui étudiait la  précision de l'échographie par rapport à l'AFP dans le  diagnostic du carcinome hépatocellulaire avec une suspicion clinique de  CHC a trouvé pour l’échographie une sensibilité de 90% et  une spécificité de 93,3% [13]. Pour un taux sérique d’AFP Z  à 500 ng/ml, ou exagérément élevé, la  spécificité était de 100% et signe le diagnostic de CHC  [13,14]. Mais aucune relation entre l’échographie et les concentrations  sériques d’AFP n’a été démontrée.  L’utilisation de l’AFP comme test de dépistage pour le CHC précoce  est remise en question en raison de sa faible sensibilité (39% à  64%) et la valeur prédictive positive (9% à 32%) malgré sa  spécificité (76% à 91%) [15]. De plus on peut observer des  taux élevés d'AFP sans qu’il existe un CHC dans les situations  suivantes : la grossesse, la mucoviscidose, une hépatite aiguëu ou chronique active ou une cirrhose et, plus rarement dans certains cancers  (gastrique, pancréatique, biliaire, testiculaire non séminomateux)  [1].

L'examen du foie cirrhotique chez quatre de nos patients  était difficile, concernant la reconnaissance des nodules de  régénération et de petits carcinomes  hépatocellulaires. Ceci est également décrit dans une  étude de Jakab [7]. Cette différenciation est d’autant plus  délicate qu’un nodule tumoral peut apparaîe au sein d’un nodule de  régénération [1].

Dans une étude  récente visant à évaluer la valeur diagnostique de  l'échographie de contraste (EDC) rapport par à échographie  Doppler et l'AFP dans le diagnostic des lésions focales hépatiques  de la cirrhose, D'Onofrio a démontré que l’EDC permettait de  caractériser de faU+00E7on précise ces lésions dans 96,6%  des cas. Cette valeur était supérieure à celles  notées pour l’échographie (72,0%), l’écho-Doppler (70,0%),  le taux de l'AFP (65,7%), la combinaison de l’échographie et de l’'AFP  (90,3%) [16]. La différence entre l’échographie et l’EDC  était statistiquement significative. L’EDC devrait donc être  utilisée pour caractériser les lésions hépatiques  focales détectées pendant la surveillance des patients  cirrhotiques. L’échographie de contraste est une technique prometteuse,  mais ses conditions de mise en oeuvre sont lourdes dans notre milieu  burkinabè.

Dans notre étude, la thrombose portale  était décrite dans 56,9% des cas. Ces chiffres sont très  proches de ceux trouvés par Llovet [17] qui rapportait une thrombose  portale dans la moitié des cas. Elle témoigne du stade  avancé de la maladie dans notre série. L’incidence de l’invasion  portale est parallèle à la taille et le nombre des tumeurs. Cette  invasion serait de 60 à 90% pour les nodules supérieurs à 5  cm et de 47% pur les tumeurs multiples [1,18].

## Conclusion

Au Burkina Faso, le CHC est de  préférence nodulaire, hyperéchogène et de taille  variable, souvent grosse, avec une forme particulière pseudo  abcédée. Parmi les options d'imagerie diagnostique,  l'échographie a un rôle de premier plan. De par ses  caractères intrinsèques, elle est d'une grande contribution dans  le diagnostic des CHC, et constitue parfois la seule approche diagnostique  d’autant plus que les examens anatomopathologiques sont souvent inaccessibles.  L’échographie peut en outre aider à améliorer le diagnostic  des CHC par le développement de la pratique des ponctions biopsiques avec  examens cytologiques et/ou histologiques qui sont plus fiables que le dosage de  l'AFP.

## Conflits d’intérêt

Les auteurs ne déclarent aucun conflit d’intérêts.

## Contribution des auteurs

Exploration échographique: Nikièma Z, Prise en charge médicale des patients: Sawadogo A et Kyelem CG, Rédaction du manuscrit: Nikièma Z et Kyelem CG.

## Patient consent

Written informed consent was obtained from the patient for publication of  this case report and a copy of it is available for review by the Editor-in-Chief  of this journal.

## Competing interests

No competing interests are involved in this case report

## Figures and Tables

**Tableau 1:** Différents aspects hépatiques observés à l’échographie

**Paramètres hépatiques**	**Nombre de cas**	**Pourcentage**
**Taille**		
Normale	4	6,9
Hépatomégalie	54	93,1
**Contours**		
Réguliers	6	10,3
Irréguliers	52	89,7
**Parenchyme**		
Homogène	4	6,9
Hétérogène	54	93,1
**Nombre de tumeur**		
Unique	16	27,6
Multiple	39	67,2
Diffuse	3	5,2
**Taille de la tumeur**		
Petite	18	31
Grande	40	69
**Echogénicité de la tumeur**		
Hyperéchogène	41	70,7
Hypoéchogène	6	10,3
Mixte	6	10,3
Pseudo suppurative	5	8,7
Cirrhose	4	6,8

**Tableau 2:** Différentes anomalies extra-tumorales observées à l’échographie

**Anomalies extra- tumorales**	**Nombre de cas**	**Pourcentage**
Ascite	35	60,3
**Thrombose ou thrombus**		
portale	35	56,8
cave inférieure	1	1,7
Adénopathies	11	18,9
Splénomégalie	6	10,3
Lithiase vésiculaire	5	8,7
Bile (sludge, boue)	13	22,4
Dilatation des voies biliaires	5	8,7
Carcinose péritonéale	2	3,4
